# How about “The effect of intraoperative cell salvage on allogeneic blood transfusion for patients with placenta accreta”? An observational study

**DOI:** 10.1097/MD.0000000000010942

**Published:** 2018-06-01

**Authors:** Kui Zeng, Wei Huang, Chao Yu, Rurong Wang

**Affiliations:** a Department of Anaesthesiology, West China Second University Hospital, Key Laboratory of Birth Defects and Related Diseases of Women and Children, Sichuan University, Chengdu, Sichuan; b Department of Anaesthesiology, West China Hospital of Sichuan University, Sichuan University, Chengdu, China.

**Keywords:** allogeneic blood transfusion, autologous blood transfusion, intraoperative cell salvage, placenta accreta

## Abstract

Intraoperative cell salvage (IOCS) for high-risk obstetric hemorrhage is now endorsed by a number of obstetric organizations. Most previous studies have focused on the safety of IOCS from case series and small controlled studies. Here, we describe the effect of IOCS on rates of allogeneic blood transfusion (ABT) under different degrees of bleeding during cesarean section in women with placenta accreta, which has seldom been reported in the literature.

We conducted a retrospective analysis on the introduction of routine application of IOCS for the management of hemorrhage during cesarean section in women with placenta accreta. We identified 115 women, with prenatally diagnosed placenta accreta/increta/percreta before this change in practice, who served as controls, and 108 women who had IOCS applied during cesarean section.

Compared with the control treatment, IOCS was significantly associated with a lower incidence of ABT (odds ratio, 0.179; 95% confidence interval, 0.098–0.328). Among the women with ≤3000 mL of bleeding, ABT was avoided in 80 (93.0%) of the 86 patients in the IOCS group, while 49 (50.0%) of the 98 controls required ABT. For women with an estimated blood loss >3000 mL, the reinfused IOCS blood may have helped prevent the need for ABT in 6 (28.6%) of the 21 patients, while all of the 17 controls required ABT. Subgroup analysis of coagulation function and the need for coagulation components showed no significant difference between the 2 groups (*P* > .05). Compared with the control treatment, IOCS was associated with a lower intraoperative volume of crystalloid (*P* < .01) and colloid infusion (*P* < .01) and a shorter length of postoperative hospital stay (*P* < .01) in patients with placenta accreta. In addition, there were no complications or adverse reactions in patients with placenta accreta who underwent IOCS.

IOCS helped reduce the need for ABT and fluid transfusion in patients with placenta accreta and may be safe for use in obstetrics.

## 1 Introduction

Over the past few decades, the incidence of placenta accreta and related conditions (i.e., placenta increta and percreta) has increased steadily from 1/4027 pregnancies in the 1970s to 1/2510 in the 1980s and 1/533 in 1982 to 2002, partly due to the rising cesarean section rate.^[1]^ The mortality rate of placenta accreta patients remains high (up to 7%) despite improvements in obstetric care.^[2]^ The average blood loss in patients with placenta accreta during cesarean section is 3 to 5 L, with 95% of patients requiring blood transfusion and 39% receiving a transfusion of 10 units or more of red blood cells (RBCs).^[3]^ Blood transfusion remains the primary solution to this problem.

Since the 1970s, intraoperative cell salvage (IOCS) technology has been widely used in many surgical specialties (vascular, orthopedic, urologic, cardiac, intracranial, and gynecological surgery). IOCS can greatly reduce the demand for allogeneic blood transfusion (ABT), and the incidence of patient-related adverse reactions is very low at only 0% to 0.006%.^[4]^ Despite some theoretical concerns regarding the application of IOCS in obstetrics, such as amniotic fluid embolism (AFE), alloimmune hemolysis caused by fetal blood, coagulation disorders, and other factors, there is increasing evidence for the safety of IOCS during cesarean section and no evidence of definite AFE occurrences associated with the use of obstetric IOCS to date.^[5–7]^ In 2013, IOCS was legally introduced in obstetric operations in the UK. However, most available clinical evidence on IOCS is from case series and small controlled studies,^[5,8–10]^ and a high-quality controlled trial with a large sample size is needed to further confirm its safety in this context. In addition, the effect of IOCS on ABT and coagulation factors for different degrees of bleeding remains uncertain.

To address this deficiency, the present study compared the amount of blood product transfusions and changes in blood routine and coagulation function in patients with placenta accreta receiving autologous and nonautologous blood transfusion. These results may serve as a guide for the optimal management of blood transfusion in placenta accreta patients undergoing IOCS.

## 2 Methods

### 2.1 Study design and patients

This study was registered at http://www.chictr.org.cn (Chinese Clinical Trials Registry number: ChiCTR-ORN-17013372). The study protocol was approved by the Ethics Committee of West China Second University Hospital. Individual consent was deemed unnecessary, as data were collected anonymously. This was a retrospective study, and data were collected from consecutively enrolled patients at our hospital with placenta accreta who had been diagnosed prenatally by ultrasound and/or magnetic resonance imaging (MRI) between August 2015 and May 2017. We searched our electronic medical record database with the following keywords: abnormal invasive placenta, placenta accreta, placenta increta, and placenta percreta. From July 2016, all women with a prenatal diagnosis of placenta accreta received IOCS. Women who had placenta accreta in the preceding 11 months from August 2015 to June 2016 served as controls. Peripartum management did not change over the study period aside from IOCS. Because IOCS was not available for emergency cesarean section, only elective cases were analyzed in this study. Each group was divided into 5 subgroups based on blood loss as follows: ≤1000, 1001 to 2000, 2001 to 3000, 3001 to 4000 mL, and >4000 mL.

### 2.2 Cell salvage

Cell Saver Elite (Haemonetics, Braintree, MA) was prepared before surgery. A 2-suction system was used to avoid contamination with amniotic fluid. The recovered blood was mixed with heparin in saline (25,000 U in 1000 mL), and the rate was adjusted to a slow drip. After processing in the automatic mode, the washed blood was pumped into blood recovery bags and transfused via the leucocyte filter (RCEZKS; Haemonetics, Braintree, MA) to the patients if necessary. If the amount of bleeding was less than 20% of systemic blood volume, the salvaged blood was transfused after hemostasis was essentially completed. ABT was considered only if the patient's hemoglobin (Hb) levels were lower than established guidelines for blood transfusion following transfer of the salvaged blood.

Women for whom cell salvage was contraindicated were excluded, including those with sickle cell disease or trait, thalassemia, or active malignancy (such as abdominal cancer). Blood aspirated from contaminated or septic wounds or surgical fields was also excluded.^[11]^ In addition, IOCS was temporarily discontinued when substances that were not licensed for intravenous use were present within the surgical field, including iodine, irrigation solutions (alcohol, betadine, or hypertonic solutions), or topical clotting agents (sponge or topical liquid forms that cause platelet aggregation, clotting activation or fibrin clot formation).

### 2.3 Fluid resuscitation

Fluid resuscitation was recommended in our hospital according to the 2009 guidelines for the prevention and management of postpartum hemorrhage published by the Scottish Obstetric Guidelines and Audit Project.^[12]^ Crystalloid solution is recommended during the initial stage of fluid resuscitation in obstetric hemorrhage. For blood loss >1000 mL and continuous bleeding or association with clinical signs of shock (hypotension, tachycardia, tachypnoea, oliguria, or delayed peripheral capillary filling), up to 3.5 L of warmed crystalloid (2 L) and/or colloid (1–2 L) should be infused as rapidly as required. Compatible blood should be transfused as soon as available, if necessary.

### 2.4 Allogeneic blood transfusion

An ABT strategy was recommended in our hospital according to 2006 guidelines for perioperative transfusion and adjuvant therapy by the American Society of Anesthesiologists and 2012 guidelines by the American Association of Blood Banks for transfusion.^[13,14]^ RBCs were infused if Hb levels were < 60 to 70 g/L and were not necessary for Hb levels >100 g/L. For Hb levels of 60 to 100 g/L, the transfusion decision was made by a physician based on a comprehensive analysis of the patient's status, including cardiopulmonary decompensation function, the degree of anemia, metabolic rate, and age.^[13,14]^ Coagulation factors were supplemented according to previous guidelines,^[12]^ which recommend maintaining prothrombin time and activated partial thromboplastin time at ≤1.5-fold normal control values, platelet count at ≥50 × 10^9^/L, and plasma fibrinogen at ≥1 g/L.

### 2.5 Cost of transfusion

At our hospital, the transfusion cost for each unit of allogeneic packed RBCs was ¥541; this included packed RBCs, ABO and Rh blood typing, antibody screening, cross matching, white blood cell (WBC) filtration, and administrative expenses. The cost of blood recovery was a flat rate charge of ¥1530, which included liner, tubing, and anticoagulant solution costs. The total cost of autologous RBCs and allogeneic packed RBCs for every patient was calculated.

According to the published methods,^[9]^ the volume of blood salvaged from each patient was converted to an equivalent number of units of allogeneic blood (PRBCeq); therefore, the cost of PRBCeq was calculated according to the current price per unit of allogeneic blood. Generally, 1 unit of allogeneic packed red cells is obtained from 200 mL of whole blood in China, which means that 200 mL of blood recovery is equivalent to 1 unit of allogeneic packed red cells. The difference between the cost of PRBCeq and IOCS corresponds to the net savings of IOCS, expressed as the following equation:

Saving = Price × PRBCeq – Cost,

where Price is the current price of per unit of allogeneic blood, and Cost is the cost of IOCS application. The savings of undergoing IOCS was calculated for each patient.

### 2.6 Data collection

Primary outcomes included the patient's volume of blood recovery and return, ABT, estimated blood loss (EBL), blood routine, and coagulation function before surgery and 24 hours after surgery. Secondary outcomes included duration of surgery and anesthesia, duration of postoperative hospital stay, urine volume, infusion volume of crystalloid and colloid, maternal adverse events, Hb level at discharge, and the cost of RBC transfusion. Perioperative fluid management was provided according to standard hospital protocols. Maternal adverse events included adverse transfusion reactions (allergy, purpura, nonhemolytic febrile reaction, bleeding, and hemolysis) and IOCS-associated adverse events (e.g., IOCS-associated hypotension and AFE-like symptoms).

### 2.7 Follow-up after discharge

In our hospital, all patients with placenta implants were required to undergo a routine follow-up at the outpatient department 2 weeks after discharge. If abnormal vaginal hemorrhage or prolonged bleeding occurred within 2 months of discharge, the patient was recommended to return to the hospital for further examination and treatment. There were no cases of rehospitalization due to postpartum hemorrhage, and no patient underwent secondary postpartum hemorrhage in either group.

### 2.8 Statistical analysis

Statistical analysis was performed using SPSS (version 20.0; SPSS Inc., Chicago, IL). Normally distributed continuous variables were reported as means (standard deviation, SD) and were compared using Student *t* tests. Non-normally distributed continuous variables were reported as medians (range) and were compared using the Mann–Whitney test (unpaired data) or Wilcoxon paired signed rank test (paired data). Regression analysis was performed to determine the association between EBL and blood recovery volume. Categorical variables were summarized as frequencies and were compared using the Chi-squared test. Statistical significance was defined as a 2-sided *P* *<* .05.

## 3 Results

### 3.1 Patient characteristics

A total of 223 eligible subjects were identified using our search criteria. These included 115 control women and 108 women who underwent IOCS, with 85 of the latter patients receiving reinfusion of IOCS blood (78.7%). The remaining 23 patients in the IOCS group did not undergo reinfusion of the salvaged blood due to an insufficient volume of recovered blood; the EBLs for these patients were all less than 1000 mL. Maternal characteristics were similar between 2 two groups, with the exception of postoperative hospital stay duration, which was shorter in the IOCS group (*P* < .01, Table 1). The rate of cesarean hysterectomy was slightly lower in the IOCS group (16.7%) than the control group (22.6%), but this marginal difference did not reach statistical significance (*P* = .31, Table 1). There were no adverse transfusion reactions or IOCS-associated adverse events in either group, and no cases of AFE-like symptoms were recorded.

**Table 1:**
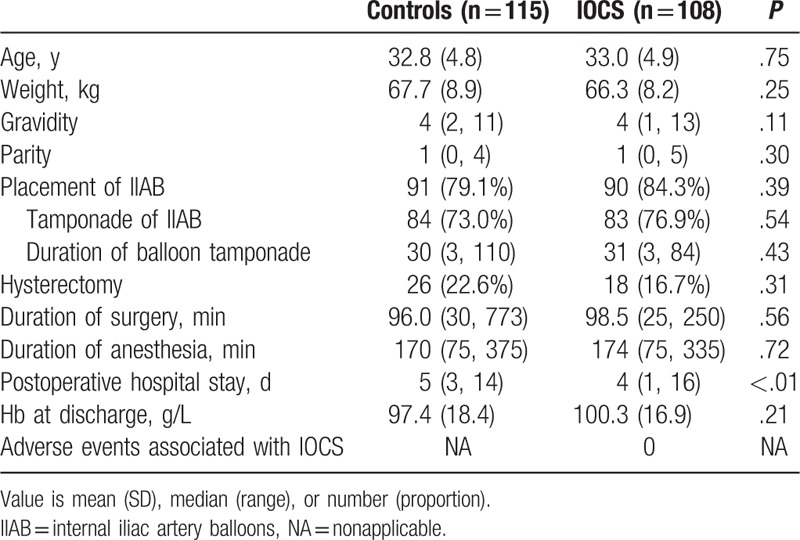
Characteristics of the patients.

### 3.2 Perioperative fluid management

**Figure 1:**
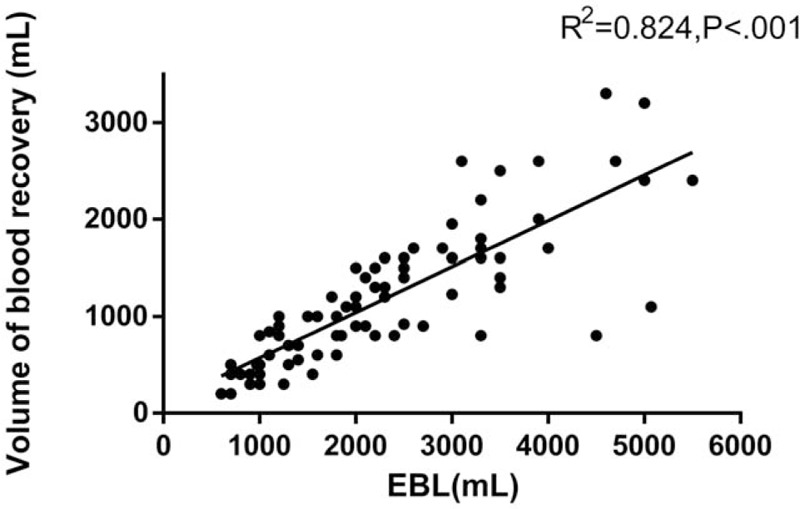
Relationship between the estimated blood loss (EBL) and the volume of blood recovery among patients reinfused IOCS blood (n = 84). Regression equation: the volume of blood recovery (mL) = 0.47 × EBL (mL) +48.85.

The use of IOCS resulted in significantly lower requirement for allogeneic RBC products (*P* < .001, Table 2), while the EBL was similar between the 2 groups (*P* = .75, Table 2). Among the 85 women receiving salvaged blood within the IOCS group, blood recovery volume was linearly correlated with the EBL (*P* < .001, Fig. 1). Compared with the control group, IOCS was significantly associated with a lower incidence of ABT (odds ratio, 0.179; 95% confidence interval, 0.098–0.328). The total allogeneic RBC transfusion volumes for all patients in the IOCS and control groups were 19,700 and 77,300 mL, respectively. The coagulation factor requirement rate was similar between the 2 groups (*P* > .05, Table 2). Crystalloid (*P* < .01) and colloid (*P* < .01) infusion volumes were significantly lower in the IOCS group (Table 2).

**Table 2:**
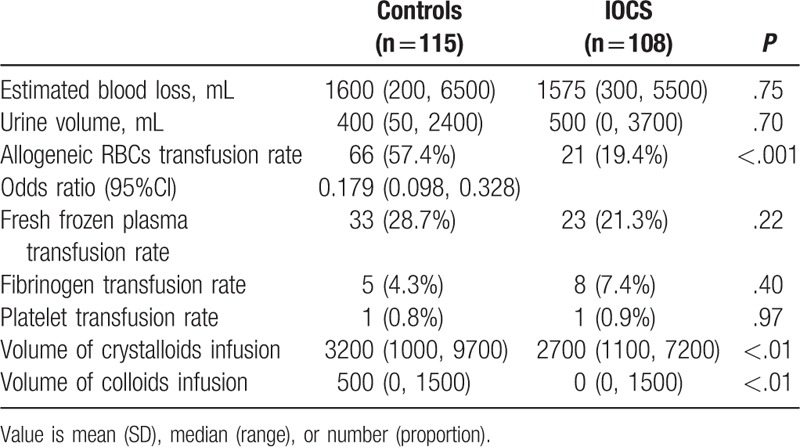
Perioperative fluid management.

### 3.3 Blood routine and coagulation function

Preoperative Hb levels for all patients were >70 g/L. The changes in blood routine and clotting function were similar between the 2 groups, except that the IOCS group showed a smaller decline in Hb levels than the control group (*P* = .02, Table 3).

**Table 3:**
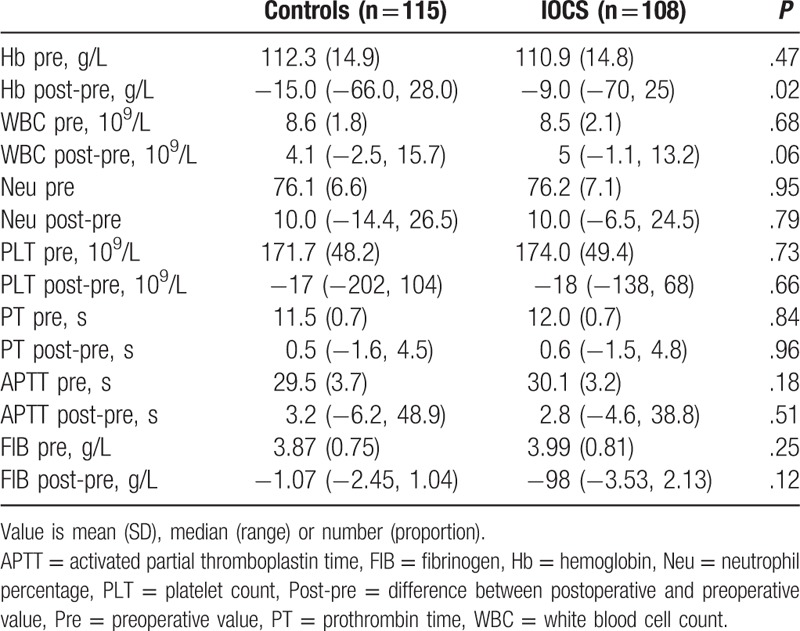
Routine blood and coagulation test of the patients.

### 3.4 Blood product transfusion volume according to the amount of blood loss

**Figure 2:**
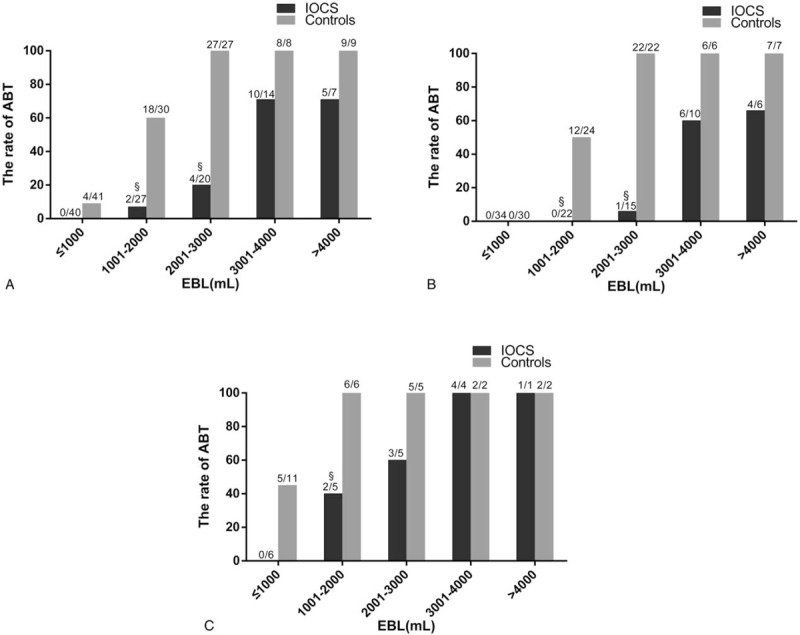
Blood product transfusion volume according to the amount of blood loss. (A) The rate of allogeneic blood transfusion (ABT) for all the patients. (B) The rate of ABT for patients with a preoperative Hb level>100 g/L. (C) The rate of ABT for patients with a preoperative Hb level ≤100 g/L. N_1_/N_2_: number of the patients required blood product transfusion/the total number of the patients according to the amount of blood loss. ^§^Compared with the control group, *P* < .05.

The constituent ratio was similar between the 2 groups (*P* = .58, Table 4). IOCS had a significant effect on the amount of ABT for all blood loss subgroups (Table 4). Among patients with blood loss >2000 mL, nearly all the patients in the control group required ABT, whereas in the IOCS group, ABT was avoided in 80.0%, 28.6%, and 28.6% of the patients in the 2001 to 3000, 3001 to 4000, and >4000 mL blood loss subgroups, respectively (Fig. 2A). Among the women in the IOCS group with a bleeding volume ≤3000 mL, ABT was avoided in 80 (93.0%) of the 86 patients, including 70 (98.6%) of the 71 women with a preoperative Hb level >100 g/L and 5 (33.3%) of the 15 women with a preoperative Hb level ≤100 g/L. In contrast, 49 (50.0%) of the 98 controls required ABT. Among the women with an EBL >3000 mL, salvaged blood reinfusion prevented the need for ABT in 6 (28.6%) of the 21 patients who underwent IOCS, while all 17 (100%) controls required ABT (Fig. 2B, C). Subgroup analysis of the demand for coagulation components showed no significant differences between the 2 groups (Tables 4 and 5).

**Table 4:**
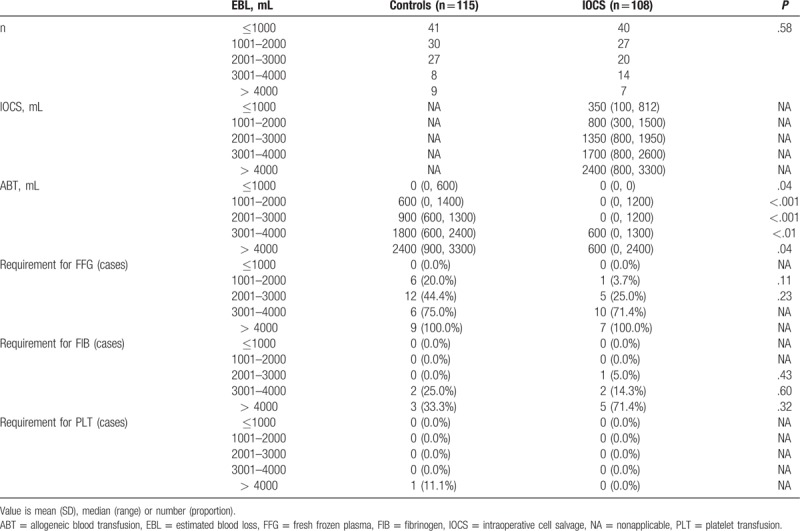
Comparison of blood product transfusion between controls and IOCS.

**Table 5:**
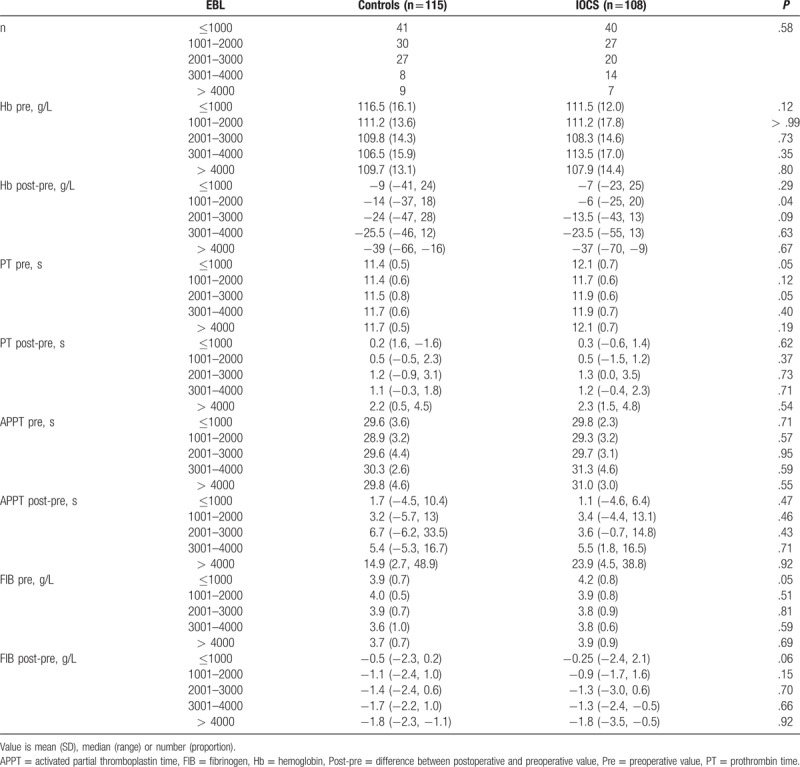
Blood routine and coagulation function according to the amount of blood loss.

### 3.5 Blood routine and coagulation function according to the amount of blood loss

Changes in Hb levels were similar between the subgroups, except that the 2001 to 3000 mL IOCS subgroup showed a smaller decline in Hb levels than the control subgroup (*P* = .04, Table 5). The analysis of coagulation function according to the amount of blood loss showed no significant differences between the 2 groups (Table 5).

### 3.6 Cost of autologous and allogeneic RBC transfusion

The cost of autologous and allogeneic RBC transfusion was calculated for each patient. According to the cost standard of our hospital, the median cost of RBC products in the IOCS group [¥1530 (1530, 8022)] was slightly lower than the control group [¥1623 (0, 8927)], but this marginal difference did not reach statistical significance (*P* = .78). Overall, the median savings resulting from IOCS application was ¥ 634 (-1153, 7397) for patients with placenta accreta during cesarean section.

## 4 Discussion

The present findings indicate that IOCS was useful for reducing the requirement of allogeneic transfusion and fluid transfusion in patients with placenta accreta during cesarean section. The requirement for clotting factor replacement was similar between the 2 groups. None of the patients in the IOCS group showed IOCS-associated adverse events. Therefore, these results support the use of IOCS in obstetric surgery.

Iatrogenic AFE is a major theoretical risk of IOCS. In this study, no AFE-like symptoms were observed. AFE is no longer considered to be caused by embolization of fetal squamous cells; rather, AFE is understood as an unknown fetal antigen-induced anaphylactoid syndrome.^[15]^ Amniotic fluid has been found to be present in healthy maternal blood during pregnancy,^[16]^ suggesting that the presence of amniotic fluid in the salvaged blood is not an absolute contraindication to IOCS during cesarean section. In previous studies, the number of fetal squamous cells in salvaged blood was comparable to that in fresh blood of healthy parturients at the time of placental separation.^[17,18]^ Through 2010, more than 400 cases used cell salvage in obstetrics.^[19]^ One obstetric death was attributed to AFE by the authors,^[20]^ but death is not a generally accepted outcome of AFE.^[19,21–26]^ Hundreds of other obstetric patients have undergone autologous blood transfusion from IOCS, and no definite case of iatrogenic AFE has been reported thus far. However, the estimated incidence of AFE is very low, affecting approximately 1.7 in 100,000 parturients in the UK.^[27]^ Therefore, tens of thousands of studies are needed to determine whether IOCS increases the risk of AFE.^[19]^

In the past, an anticipated blood loss ≥20% of total estimated blood volume or transfusion requirements for >10% of patients undergoing a given procedure were considered indications for cell salvage by the American Association of Blood Banks.^[28]^ The use of cell salvage for cases at high risk of obstetric hemorrhage has been reported to be economically reasonable.^[29]^ In our study, 42.6% (49 of 115) of controls with placenta accreta during cesarean section required ABT. Therefore, placenta accreta may be a good indication for the application of IOCS. As in previous studies, IOCS in patients with placenta accreta was found to reduce the need for ABT.^[7]^ ABT was avoided in 70 (98.6%) of the 71 women with a blood loss volume ≤3000 mL and a preoperative Hb level>100 g/L. Thus, IOCS almost completely prevented the need for ABT in patients with preoperative Hb levels >100 g/L and blood loss ≤ 3000 mL. However, for placenta accreta patients with an EBL exceeding 3000 mL, IOCS may not obviate the need for RBC transfusion, as 71.4% of these patients in the IOCS group still received ABT. The need for ABT during the procedure is largely dependent on blood recovery as a percent of EBL. Our study showed that the blood recovery volume was approximately 47% of the EBL, which indicated that more than 50% of the EBL could not be recovered by IOCS. Postpartum hemorrhage may be severe because of increased uterine artery blood volume flow, from 513 mL/min (SD, 127 mL/min) at 20 weeks to 970 mL/min (SD, 193 mL/min) at 38 weeks.^[30]^ The probability of allogeneic RBC transfusion may increase significantly in patients who experience excessive and rapid postpartum hemorrhage. An extra suction system for IOCS should be considered in acute massive hemorrhage, as the use of only 1 suction system may not be sufficient to collect sufficient blood in a timely and effective manner. In addition, some bleeding was discharged from the vagina or was absorbed by cotton pads, which could not be recycled during cesarean section. Intraoperative bleeding without amniotic fluid should be retained by IOCS rather than absorbed by cotton pads as much as possible. If blood loss exceeds 3000 mL, extra attention should be given to changes in Hb levels, and allogeneic RBCs should be transfused if necessary while implementing IOCS.

The requirement for clotting factor replacement was similar between the 2 groups. Heparin/citrate contamination, which had been present in autologous blood, is now completely removed with new techniques.^[22]^ After washing, coagulation components (e.g., plasma, platelets, and coagulation factors) are mostly removed, with RBCs remaining in the recovered blood. Coagulation disorders have been considered a theoretical risk associated with the use of IOCS. Rudra and Basak^[31]^ reported 1 patient who underwent IOCS and experienced intraoperative coagulopathy during major obstetric hemorrhage. Postsurgical thrombin time and prothrombin time have been reported to be lower in some IOCS subgroups in patients with ectopic pregnancy,^[32]^ while the infusion rate of coagulation components was consistently higher in IOCS groups in this study. It was difficult to judge whether the increase of thrombin time and prothrombin time was caused by the use of IOCS or by the different coagulation component infusion rates. In our study, the requirement for clotting factor replacement and change in coagulation function were similar between the autologous and ABT groups. We think that coagulation disorders are a potential complication associated with hemorrhage and secondary dilutional coagulopathy but not with cell salvage itself. Whether allogeneic or autologous blood is transfused, the requirement for coagulation component replacement is entirely dependent on the volume of clotting factors lost.

The volume of crystalloid and colloid infusions was somewhat lower in the IOCS group than the control group. When patients need blood transfusion therapy due to postpartum hemorrhage, crystalloid or colloid may temporarily substitute for a blood transfusion to maintain circulating blood volume while waiting for cross-match testing and the acquisition of blood products from the blood bank. For patients who undergo IOCS, autologous blood can be collected at the bedside and immediately reinfused into the patients. Thus, IOCS had no significant effect on the requirement for the substitutive crystalloid or colloid infusion in patients with placenta accreta.

The duration of postoperative hospital stay was significantly shorter in the IOCS group, suggesting that IOCS resulted in good clinical outcomes. Huang et al^[32]^ reported that cell salvage transfusion did not lead to adverse reactions or complications and resulted in shorter hospitalization stay. Studies comparing cell salvage with ABT have found increased oxygen delivery efficacy and mean erythrocyte viability in salvaged blood.^[24,33–36]^ The mean erythrocyte viability with cell salvage has been reported to be as high as 88%.^[37]^ Therefore, oxygen-carrying capacity and tissue oxygen delivery are posited to be improved with cell salvage. The physiological properties of salvaged blood may contribute to the shorter duration of postoperative hospital stay. This economic benefit may be another potential advantage of IOCS.

The main limitation of our study is its retrospective design. Selection bias may exist for the subjects who were not randomly allocated. However, we performed a thorough case assessment, and the 2 groups were well balanced. In addition, there are no clear guidelines for autologous blood transfusion in our country. Thus, when Hb levels do not meet the criteria for ABT, physician judgment is used to determine whether the salvaged blood should be retransfused. Additional studies are necessary to definitively determine whether IOCS increases the risk of AFE.

## 5 Conclusion

The present findings support the use of IOCS to reduce the need for ABT in placenta accreta patients. These results serve as a reference and evidence for the management of blood transfusion in placenta accreta patients undergoing IOCS.

## Acknowledgments

The authors thank Mr. Tianqiang Qin for his statistical analysis of the study data. We would also like to thank American Journal Experts (http://www.aje.cn) for English language editing.

## Author contributions

**Conceptualization:** Kui Zeng.

**Data curation:** Kui Zeng, Wei Huang, Chao Yu, Rurong Wang.

**Formal analysis:** Kui Zeng, Wei Huang.

**Investigation:** Kui Zeng, Wei Huang, Chao Yu.

**Methodology:** Kui Zeng, Wei Huang, Chao Yu, Rurong Wang.

**Project administration:** Kui Zeng, Wei Huang.

**Software:** Kui Zeng, Rurong Wang.

**Supervision:** Wei Huang, Rurong Wang.

**Writing – original draft:** Kui Zeng, Rurong Wang.

**Writing – review & editing:** Kui Zeng, Rurong Wang.
